# COVID-19 long: evaluation of quality of life, sarcopenia and proteinuria

**DOI:** 10.11606/s1518-8787.2025059006122

**Published:** 2025-10-17

**Authors:** Sayane Marlla Silva Leite Montenegro, Roberto Marcó, Marília de Almeida Correia, Rosilene Motta Elias, Maria Aparecida Dalboni

**Affiliations:** I Instituto Federal de Educação, Ciências e Tecnologia de Pernambuco. Departamento de Enfermagem. Belo Jardim, PE, Brasil; II Universidade Nove de Julho. São Paulo, SP, Brasil; III Universidade Nove de Julho. Programa de Pós-Graduação em Ciências da Reabilitação. São Paulo, SP, Brasil; IV Universidade Nove de Julho. Programa de Pós-Graduação em Medicina. São Paulo, SP, Brasil

**Keywords:** COVID-19, Quality of Life, Sarcopenia, Proteinuria

## Abstract

**OBJECTIVE:**

To evaluate quality of life, sarcopenia and proteinuria, six and 12 months after infection with mild and moderate COVID-19.

**METHODS:**

We evaluated 253 individuals with mild (n = 119) and moderate (n = 134) clinical presentation for COVID-19 (reverse transcription-polymerase chain reaction—RT-PCR) after six (T6) and 12 (T12) months from the date of acute infection (T0). Quality of life, pain, risk for sarcopenia, muscle strength and proteinuria were assessed by the Short Form Health Survey 36 (SF-36) questionnaire; visual analogue scale (VAS); the Simple Questionnaire to Rapidly Diagnose Sarcopenia (SARC-F); hand grip and sit-up and the urinalysis strip, respectively.

**RESULTS:**

The average age was 44 ± 10 and 43 ± 12 years; female 68 and 59% for the mild and moderate groups, respectively. Seventy-five percent or more of patients were vaccinated with at least two doses before acquiring COVID-19 infection. Individuals with a moderate clinical presentation in relation to mild cases were hypertensive (23 and 6%, p < 0.001) and had diabetes mellitus (9 and 2%; p = 0.01) at the time of COVID-19 acute infection. The moderate group at T6 presented lower functional capacity (SF36: 46 ± 20 vs. 61 ± 24); more pain (SF36: 45 ± 29 vs. 67 ± 32 and VAS: 55 vs. 32%); greater dysfunctionality for daily activities (Duke Activity Status Index—DASI: 40 ± 11 vs. 45 ± 10); lower limb muscle strength (sit-up: 9 ± 2 vs. 11 ± 2); higher risk for sarcopenia (SARC-F: 6 ± 4 vs. 4 ± 3) and higher proteinuria ≥ 1"+": 59 vs. 42%) compared to the mild group. After 12 months, the moderate group remained with greater pain (SF36+VAS) and more dysfunctionality in daily activities (DASI) compared to the mild group.

**CONCLUSION:**

Comparing T12 to T6, we observed that the mild group had worse functional capacity; more pain (SF36+VAS); lower upper limb strength and higher proteinuria ≥ 1"+": 63 vs. 42%). We observed a negative correlation between SARC-F score and sit-up; functional capacity (SF36).

## INTRODUCTION

In the period from December 2019 to June 2024, approximately 700 million cases of COVID-19 were confirmed worldwide: approaching 6 million deaths^1^. In Brazil, one of the countries most affected by the pandemic, there were more than 39 million cases and around 800 thousand deaths^1^. COVID-19 has manifested with several systemic disorders, particularly severe pneumonia and acute respiratory distress syndrome^2^. In most individuals, COVID-19 presented with a mild, moderate or asymptomatic clinical presentation according to the World Health Organization (WHO) classification, based on the clinical condition presented by the patient at the time of infection^3,4^.

COVID-19 is caused by the SARS-CoV-2 virus^5^. The clinic involved in COVID-19 is complex because it is not specific, considering that the virus binds to the Angiotensin Converting Enzyme 2 (ACE2) receptor, present in the membranes of various types of cells in different organs^6^. The link of SARS-CoV-2 to ACE2 receptor signalizes an acute inflammatory response, including cytokine production, which results in deleterious effects on organs^5^. Thus, COVID-19 presents itself as a systemic infectious disease characterized by a severe inflammatory state^6^.

Numerous sequelae of COVID-19 are being reported in relation to critically ill patients. Chakraborty and Maity^7^ observed that 15% of hospitalized COVID-19 patients had at least some renal alteration assessed by an increase in the serum concentration of urea and creatinine or a reduction in the estimated glomerular filtration rate (eGFR). Other studies have observed mild proteinuria (30%) and hematuria (30%) in critically ill patients^8,9^. In a multicenter study, one in six patients required dialysis within 60 days of admission to the intensive care unit (ICU) and one in three patients required dialysis after hospital discharge^10^. In addition to renal issues, sequelae such as depletion of skeletal muscle mass and muscle strength have been described^9^.

The WHO estimated that 10 to 20% of patients recovered from severe COVID-19 had persistent symptoms for months after infection and recognized that this condition has a direct impact on the individual's health, quality of life, in addition to increasing healthcare costs to the economy and productivity^11^.

However, there are few if any studies evaluating pain, risk of sarcopenia, muscle strength, functionality for daily activities, quality of life and renal function in patients with long COVID-19 who had mild and moderate clinical presentation at the time of COVID-19 acute infection. Thus, the objective was to evaluate quality of life, sarcopenia and proteinuria, six and 12 months after infection with mild and moderate COVID-19.

## METHODS

### Study Design

This is a quantitative, longitudinal study that initially evaluated 550 medical records of individuals who had COVID-19 (positive reverse transcription-polymerase chain reaction—RT-PCR) treated at the Reference Center for COVID-19, a reference unit for COVID-19 in São Gabriel da Cachoeira/AM, Brazil, from December 2021 to January 2022. São Gabriel da Cachoeira is a Brazilian municipality in the state of Amazonas, in the northern region of the country, located more than 800 km from the capital. São Gabriel da Cachoeira belongs to the Alto Rio Negro Region, with a population of 51,795 inhabitants, bordering Colombia and Venezuela. Health coverage in the city is provided by the Alto Rio Negro Special Indigenous Health District (DSEI-ARN), the Municipal Health Department and the Brazilian Army through the Garrison Hospital. The city therefore has a primary and secondary care network. When a tertiary or specialized network is needed, patients are transferred to Manaus/AM.

The questionnaires were applied and the biological sample was collected at the COVID-19 Reference Center between January 2022 and January 2023 by the researcher involved, who is also a nurse. There was no sample loss due to death, dropout or lack of follow-up. In this sense, participants were followed up at six and 12 months after infection with COVID-19. Convenience sampling was used.

**Figure 1:**
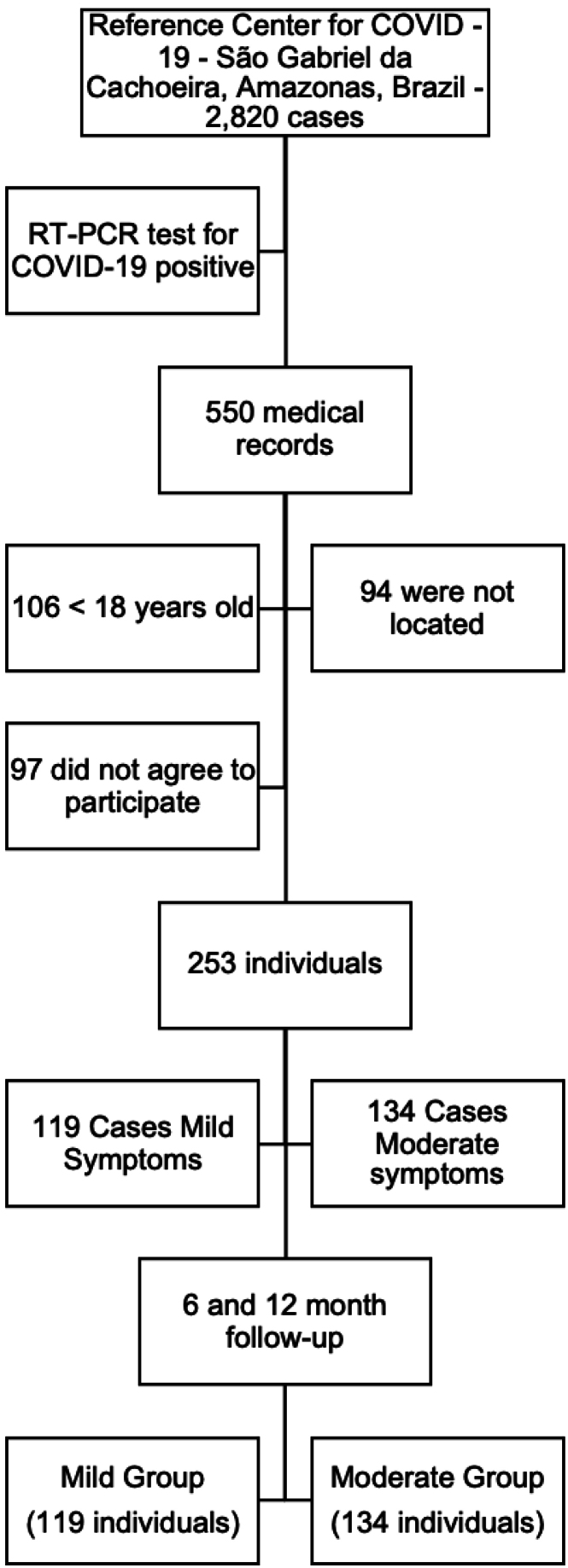
Study flowchart from selection and inclusion of individuals.

Of the 550 individuals, 253 individuals with mild (n = 119) and moderate (n = 134) clinical presentation for COVID-19 (positive RT-PCR) after six (T6) and 12 (T12) months from the date of acute infection (T0) and that completed the 6- and 12-month follow-up period were included in the study (Figure 1), according to the following criteria. *Inclusion criteria*: adult individuals (≥ 18 years old), who had mild and moderate clinical presentation at the time of infection, according to the criteria of the Ministry of Health/Brazil (20) and which were evaluated six and 12 months after COVID-19 infection. *Non-inclusion criteria:* patients who underwent surgery on the arm or hand in the three months prior to data collection, who were hospitalized due to a severe clinical presentation requiring intensive care and individuals with post-intensive care syndrome who did not complete the time follow-up.

The 253 individuals were evaluated six and 12 months after the date of COVID-19 acute infection for demographic, clinical data, proteinuria (urinary strip) and applied Visual Analogue Scale for pain (VAS), Short Form Health Survey 36—SF-36 (quality of life assessment), plantar pressure strength (hand grip/digital dynamometer), Simple Questionnaire to Rapidly Diagnose Sarcopenia (SARC-F) scale and sit-up test. The VAS, SF-36 and SARC-F questionnaires were used to assess self-reported functional capacity, pain and skeletal muscle mass post-COVID-19 infection.

### Data Collection

Eligible individuals were contacted and invited to answer the questionnaires and have the biological samples collected at the Reference Center for COVID-19 in São Gabriel da Cachoeira/AM. It should be remembered that data collection at the COVID-19 center followed all recommendations from the Ministry of Health regarding the pandemic, such as: use of masks, distancing, use of 70% alcohol for hand hygiene and equipment before and after measurements, as well as complete immunization (anti-COVID19 vaccine) by the researcher.

### Characterization of Mild and Moderate Clinical Presentation, According to the Criteria of the Ministry of Health/Brazil

Individuals were stratified into mild and moderate clinical presentation according to the COVID-19 classification, based on the criteria established by the Ministry of Health (2020)^12^:

- Epidemiological and demographic data: Demographic data, Quantitative reverse transcription polymerase chain reaction (RT-qPCR) results, clinical classification of COVID-19, comorbidities, clinical evolution of acute infection and dates of onset of symptoms and vaccination were collected from the electronic medical record of the Reference Center for COVID-19, São Gabriel da Cachoeira/AM, Brazil, in times: T0, T6 and T12;
- Duke Activity Status Index questionnaire: The DASI questionnaire contains 12 questions about the functionality of the individual's daily and common activities. This questionnaire was developed on a sample of American adults self-reporting their daily activities^13^ and has a score that varies from 0 to 58.2, where the higher the score, the greater the individual's functionality;
- Pain Visual Analog Scale: The VAS establishes a numerical value in relation to how much pain the individual feels, and the patient can demonstrate their feeling of pain through images (the images have a scale from 0 to 10);
- Short Form Health Survey 36 questionnaire: The SF-36 questionnaire is an instrument that assesses quality of life, encompassing functional capacity, physical aspects, pain, general state of health, vitality, social aspects, emotional aspects, relationship between health and work, activities of daily living and mental health, and has been used to assess patients with COVID-19^14^. The score ranges from 0 = worst to a maximum of 100 = best for each domain (Gross Scale). According to the aim of this study, only functional capacity, physical aspects and pain were analyzed;
- Simple Questionnaire to Rapidly Diagnose Sarcopenia questionnaire: The SARC-F^15^ questionnaire is widely used to measure the risk of sarcopenia, where five components are assessed: strength, walking assistance, getting up from a chair, climbing stairs and falls in the last six months. The questionnaire used was the one that includes calf measurements. The total SARC-F score ranges from 0 to 20, with individuals with a score ≤ 3 being at low risk; ≥ 4 some risk and ≥ 8, high risk for sarcopenia. In this study, we considered the risk of sarcopenia to be ≥ 4;
- Sit-up Test: The Sit and Stand Test (SST) makes it possible to quickly assess the flexibility of lower limb joints, lower limb strength, balance, motor coordination, the relationship between muscle power and body weight, while also characterizing minimal functional muscle fitness. The test was carried out over 30 seconds (short SST), using a chair with a standard height of 46 – 48 cm positioned against a wall, where the participant was asked to perform sitting and standing movements^16^. For 30 seconds, the participant sat down and stood up from the chair repeatedly and as quickly as possible. ≥15 repetitions in 30 seconds is considered a normal assessment standard^17^.

### Palm Grip Strength Test (Hand Grip)

Peripheral muscle strength was assessed based on hand grip strength using digital dynamometry (Saehan dynamometer, São Paulo, Brazil), in accordance with the recommendations of the American Society of Hand Therapists. Participants were properly oriented and positioned to sit without support with knees flexed at 90°, with an upright posture and the elbow flexed at 90° next to the trunk. To carry it out, individuals were asked to use the strength of their dominant and non-dominant hand to exert force by pressing the device, generating a plateau of 5 seconds. The process was then repeated three times for the dominant hand, to obtain the most acceptable and reproducible measurement. Handgrip strength is considered a normal assessment standard of < 16 kgf for women and < 27 kgf for men^17^.

### Assessment of Proteinuria

Proteinuria was assessed using a colorimetric test on a urinalysis strip (Uriquest plus, Labtest), due to the impossibility of carrying out other techniques. The test was carried out on the first urine of the morning (concentrated for 8 hours), not centrifuged and kept between 15 and 25°C. The urine was collected in a sterilized bottle and the subjects were instructed not to perform any exercises that required effort before collecting the urine.

The tape was removed individually at the time of analysis and placed in contact with the individual's urine for 60 seconds, according to the manufacturer's instructions. The test detects values equal to or greater than 30 mg/dL. In the presence of proteinuria, there is a coloration proportional to the protein concentration^18^. The intensity of the color was indicated as: absence, +, ++ and +++ (crosses). The reading was always taken by the same individual. In this study, we considered proteinuria ≥ 1 to be "+".

### Statistical Analysis

The normality of the variables was verified using the Kolmogorov-Smirnov test. Categorical variables were presented as absolute value and percentage. The comparison of continuous variables according to the classification of mild and moderate COVID-19 presentation were evaluated using Student's t-test (parametric data) and categorical variables using the χ^2^ test. The paired t-test was used to evaluate continuous variables for the groups (mild and moderate) and categorical variables using the McNemar test for six and 12 months. P < 0.05 were considered statistically significant. The analyses were carried out using the Statistical Package for the Social Sciences (SPSS) version 28.0 software.

## RESULTS

Demographic, clinical and epidemiological data at T0 are listed in Table 1. Of the 253 individuals, 119 (47%) had a mild and 134 (53%) moderate clinical presentation. There was no difference in age and sex between the groups. Individuals with moderate clinical presentation had more hypertension and diabetes mellitus. More than 75% of individuals for both groups were vaccinated with two doses of the vaccine for COVID-19. There was no difference in the type of immunizer between the groups (Table 1).

**Table 1:** Epidemiological and clinical data from 550 eligible participants and the 253 participants selected and included in the study according to mild (n = 119) and moderate (n = 134) clinical presentation of COVID-19 in the time of acute infection (T0).

COVID-19 (T0)					
		n 550	Mild (n 119)	Moderate (n 134)	P-value
Age (years) (m/DV)		38.8 (12.5)	44 (10)	43 (12)	0.70
Sex (female) (%)		319 (58)	81 (68)	79 (59)	0.13
Race (white) (%)		242 (44)	79 (66)	82 (61)	0.39
Body Mass Index (BMI) (%)					0.21
	> 18.5 to 24.9 kg/m^2^	149 (27)	30 (25)	28 (21)	
	≥ 25 to 29.9 kg/m^2^	239 (44)	56 (47)	55 (41)	
	> 30.0 kg/m^2^	162 (29)	33 (28)	51 (38)	
Diabetes Mellitus (%)		19 (3.5)	2 (2)	12 (9)^a^	0.01
Hypertension (%)		51(9.3)	7 (6)	31(23)^a^	< 0.01
Cardiovascular Disease (%)		8 (4.3)	0/0	4/3	0.06
**Vaccination (Doses) (%)**					0.01
	0 – 1 dose		16 (13)	34 (25)	
	≥ 2 doses		103 (87)	100 (75)	

DV: desvio de valores. BMI < 18.5: underweight, BMI ≥ 18.5 and < 25.0: (eutrophic), BMI ≥ 25.0 and < 30.0: overweight, BMI ≥ 30.0: obesity.

a p χ^2^.

Individuals in the moderate group had worse quality of life assessed by the domains of functional capacity and pain (SF-36), greater pain (VAS), greater dysfunctionality for daily activities (DASI) and higher score for sarcopenia risk compared to the mild group in T6. We observed that at T6 the moderate group had lower limb strength compared to the mild group assessed by the sit-up test.

At T12, the moderate group had more pain than the mild group and the mild group also had more pain compared to T6. The moderate group showed greater dysfunctionality for daily activities (DASI) compared to the mild group and lower limb strength (HPP) and improved sarcopenia risk score at T12 compared to T6 (Table 2).

**Table 2:** Assessment of quality of life (SF-36 for functional capacity and pain), pain scale (VAS), functionality (DASI) and muscular strength (palmar pressure strength [PPS], sit-up test [sit-up]) and risk of sarcopenia (SARC-F) from 253 participants six and 12 months after acute COVID-19 infection according to the COVID-19 classification symptomatology.

COVID-19									
		T6			T12			P-value	
		Mild (n = 119)	Moderate (n = 134)	p^b^ (Group)	Mild (n = 119)	Moderate (n = 134)	p^b^ (Group)	Time Mild (^c^)	Time Moderate (^d^)
Quality of life									
	SF-36 – Functional Capacity (m/DV)	61 (24)	46 (20)^b^	< 0.0001	52 (19)^c^	50 (20)	0.65	0.001	0.08
	SF-36 – Pain (m/DV)	67 (32)	45 (29)^b^	< 0.0001	58 (26)^c^	45 (21)^b^	< 0.0001	< 0.0001	0.78
Pain Scale (VAS)									
	≥ 5 (n/%)	38/32	74/55^b^	< 0.0001	48/40^c^	87/63^b^	< 0.0001	< 0.0001	0.17
Functionality and muscular strength									
	DASI (score) (m/DV)	45 (10)	40 (11)^b^	< 0.0001	43 (10)	41 (12)^b^	< 0.0001	0.14	0.40
	PPS (m/DV)	36 (18)	33 (20)	0.29	29 (14)^c^	29 (16)^d^	0.95	0.001	0.04
	Sit-up (m/DV)	11 (2)	9 (2)^b^	0.001	10 (2)	11 (2)^b,d^	0.03	0.20	< 0.0001
Risk of sarcopenia									
	SARC-F (score average) (m/DV)	4 (3)	6 (4)^b^	< 0.001	4 (3)	4 (4)^d^	0.44	0.24	< 0.0001
	SARC – F (score ≥ 4) (n/%)	81/68	57/43^b^	< 0.0001	70/59	83/62^d^	0.61	0.15	0.003

p^b^: moderate ≠ mild; p^c^: mild group T12 ≠ T6; p^d^: moderate group T12 ≠ T6. SF-36 Functional Capacity and Pain (Raw Scale: 0 = worst and 100 = best for each domain). Pain Scale (VAS) – 0 none and 10 greatest pain. DV: desvio de valores. DASI – total score ranging from 0 to 58.2, as the higher the score, the greater the functionality. PPS: reference value = women < 16 kgf and men < 27 kgf. Sit-up: reference value ≥ 15 repetitions in 30 seconds. SARC-F – score ≥ 4 indicates risk of sarcopenia.

According to Table 3, we observed that proteinuria was higher in the moderate group compared to the mild group at T6 (p = 0.01). These differences remained after excluding patients who had hypertension and diabetes mellitus. The mild group presented a greater number of cases with proteinuria at T12 compared to T6.

**Table 3:** Proteinuria data according to the mild/moderate groups and excluding patients with diabetes mellitus and hypertension from participants six and 12 months after COVID-19.

Proteinuria								
	T6			T12			*P*	
	Mild (n = 110)	Moderate (n = 97)	Group (^a^p)	Mild (n = 110)	Moderate (n = 97)	Group (^b^p)	Mild time (p^c^)	Moderate Time (p^d^)
			0.02			0.20	0.01	0.03
Absent (n (%))	64 (58)	39 (40)		41 (37)	48 (49)			
1+ (n/%)	41 (37)	45 (46)^b^		59 (54)^c^	42 (43)			
2+ (n/%)	5 (5)	13 (13)^b^		10 (9)^c^	7 (7)^d^			

p^a^ χ^2^: ^b^: moderate ≠ mild; ^c,d^McNemar: ^c^T12 ≠ T6 for mild group and ^d^T12 ≠ T6 for moderate group.

**Figure 2:**
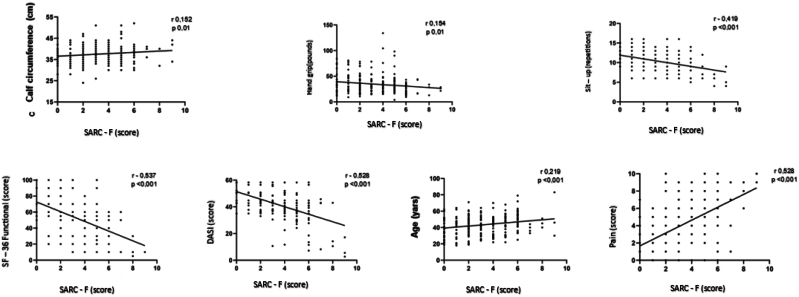
Correlation between SARC-F (score) and calf circumference (cm), hand grip (pounds), sit-up (repetitions), functional SF-36 (score), DASI (score), age (years) and pain (score) for the 253 individuals followed for 12 months, with biannual assessments. Pearson correlation test, p < 0.05.

We observed that individuals who had a higher SARC-F score showed a negative correlation with upper limb strength (handgrip/palmar pressure strength—PPS); lower limbs (sit-up); functional capacity (SF-36); dysfunction for daily activities (DASI) and positive correlation with age and pain (Figure 2).

## DISCUSSION

Several studies have described that critically ill patients have greater sarcopenia due to acute inflammation caused by a higher concentration of inflammatory cytokines in response to the presence of COVID-19^7-10^. Decreased muscle mass has been observed not only in acute SARS-COV2 infection, but also in prolonged COVID-19 in these patients^19-21^. Sarcopenia in severe COVID-19 has resulted in sequelae such as pain, lower functional capacity, lower muscle strength; associated with deficits in daily activities and poorer quality of life^22^.

However, for the first time, our study describes these sequelae in individuals who had a mild or moderate presentation of COVID-19. We observed a higher risk of sarcopenia (SARC-F) and reduced lower limb strength (sit-up) in individuals in the moderate group, especially after six months of COVID-19 infection. Handgrip strength and the sit-up test are instruments commonly used to assess muscle strength. Although we did not observe a significant difference between the groups for handgrip, both showed a decline in limb strength at T12 compared to T6. In addition, both individuals with a mild or moderate condition had lower dominant strength values compared to the reference values for this test (≥ 16 for women and 27 for men) according to age and gender^19-21^.

Decreased muscle strength, as well as the risk of sarcopenia, are associated with a higher likelihood of adverse outcomes in the general population, including falls, fractures, physical disability, decreased ability to perform activities of daily living, and mortality^23^.

Our study observed a correlation between the risk of sarcopenia (SARC-F score) and worse upper limb strength (handgrip) and lower limb strength (sit-up); worse functional capacity (SF-36); greater dysfunction for daily activities (DASI) and more pain; suggesting that COVID-19, even in the non-severe population, may also have an adverse impact on muscle mass and be associated with a worse quality of life. It is possible that the inflammatory cytokine storm that is also present in high concentrations at the time of infection in individuals with mild and moderate presentation^24^ contributes to catabolism and deterioration of skeletal muscle mass, even after recovery from the infection, since inflammation in this population has been described to persist for eight months after infection^25^. However, this is a hypothesis, since pro-inflammatory cytokines were not evaluated in the present study.

In addition to pain, sarcopenia and poorer quality of life, we also observed proteinuria in both groups, being higher in the group with moderate clinical presentation at T6. This proteinuria remained in individuals with moderate clinical presentation at T12 and increased among patients with mild clinical presentation.

Acute kidney injury was also an important complication in critically ill patients in the acute phase of SARS-COV-2 infection. Some studies report that 36.6 to 56.9% of hospitalized patients^11,16^ had at least some renal alteration assessed by an increase in serum urea and creatinine concentration or a reduction in eGFR 38 and some required dialysis during their stay in the ICU and after recovery^18,24,26^. Huart et al.^25^, in a retrospective and observational study, evaluated the renal function of 153 patients hospitalized with severe COVID-19 through total proteinuria (computerized method according to the Kidney Disease: Improving Global Outcomes—KDIGO organization) and observed that 14% of patients had proteinuria < 150 mg/g of urinary creatinine, 42% between 150 and 500 mg/g and 44% above 500 mg/g).

Our study evaluated proteinuria through the urinalysis strip test and observed that both groups with mild and moderate clinical presentation of COVID-19 presented ≥ 1"+" of proteinuria at six and 12 months and that these results were maintained after exclusion of individuals who had hypertension and diabetes. To date, there are no studies that have evaluated proteinuria in this population in long COVID-19, especially in mild and moderate cases. Hong D et al.^26^ observed 30 and 40% of proteinuria in this population, using an automated method, but at the time of infection.

Although our study population is not critically ill patients, the observed presence of proteinuria in long COVID-19 (up to 12 months after infection) may suggest that the presence of the SARS-CoV-2 virus in individuals with mild and moderate presentation may have caused some damage to the glomerulus. However, to discern whether this damage was transient or not, or whether it had an impact on renal filtration, it is necessary to evaluate other serum markers that are more sensitive to assess this function.

*As a* limitation, it was not possible to obtain proteinuria or creatinine results from individuals at T0; we used self-reported questionnaires for quality of life, pain, and risk of sarcopenia; we did not evaluate markers or imaging studies to assess sarcopenia and biomarkers of inflammation to determine their association with sarcopenia. However, this is one of the rare studies in individuals with long COVID-19 who had mild and moderate presentation and that assessed quality of life, sarcopenia, and proteinuria.

Even with the limitations observed, the study serves as a parameter for better targeting of primary health care actions and a comprehensive look at the patient who had COVID-19, as well as directing managers to mitigate adverse events through rehabilitation and quality of life programs.

## CONCLUSIONS

Our findings show that individuals with mild and moderate COVID-19 who have recovered from the infection have reduced functionality for daily activities, greater pain after six and 12 months, higher risk of sarcopenia, and reduced muscle strength, which may contribute to worsening the quality of life of these individuals. In addition, the presence of proteinuria in this population is an alert to evaluate renal function through other biomarkers, as well as to determine whether this proteinuria is transient or whether the infection caused by COVID-19 may be a new risk factor for the disease, loss of renal function and progression of kidney disease. Therefore, it is necessary to develop strategies for physical rehabilitation and follow-up renal evaluation in individuals who had COVID-19 with mild and moderate clinical presentation.

## Data Availability

The datasets generated and/or analyzed during the present study are not publicly available due to [ethical/legal/privacy] restrictions, but are available from the corresponding author upon request.
